# Redox Regulation of Mitochondrial Potassium Channels Activity

**DOI:** 10.3390/antiox13040434

**Published:** 2024-04-03

**Authors:** Joanna Lewandowska, Barbara Kalenik, Antoni Wrzosek, Adam Szewczyk

**Affiliations:** Laboratory of Intracellular Ion Channels, Nencki Institute of Experimental Biology, Polish Academy of Sciences, 02-093 Warsaw, Poland; j.lewandowska@nencki.edu.pl (J.L.); b.kalenik@nencki.edu.pl (B.K.); a.wrzosek@nencki.edu.pl (A.W.)

**Keywords:** mitochondria, reactive oxygen species, mitochondrial potassium channel, redox, ischemia/reperfusion injury, potassium, calcium, ROS

## Abstract

Redox reactions exert a profound influence on numerous cellular functions with mitochondria playing a central role in orchestrating these processes. This pivotal involvement arises from three primary factors: (1) the synthesis of reactive oxygen species (ROS) by mitochondria, (2) the presence of a substantial array of redox enzymes such as respiratory chain, and (3) the responsiveness of mitochondria to the cellular redox state. Within the inner mitochondrial membrane, a group of potassium channels, including ATP-regulated, large conductance calcium-activated, and voltage-regulated channels, is present. These channels play a crucial role in conditions such as cytoprotection, ischemia/reperfusion injury, and inflammation. Notably, the activity of mitochondrial potassium channels is intricately governed by redox reactions. Furthermore, the regulatory influence extends to other proteins, such as kinases, which undergo redox modifications. This review aims to offer a comprehensive exploration of the modulation of mitochondrial potassium channels through diverse redox reactions with a specific focus on the involvement of ROS.

## 1. Introduction

A redox (reduction-oxidation) reaction is a chemical process in which the oxidation state of atoms or their groups changes. These reactions play a crucial role in various fundamental cellular functions. Both photosynthesis and cellular respiration involve complex redox reactions. In cellular respiration, redox reactions occur as glucose, fatty acids, and proteins undergo oxidation to form carbon dioxide, while oxygen is concurrently reduced to water [1,2,3,4].

Redox reactions also manifest when reactive oxygen species (ROS) or nitrogen reactive species (RNS) impact molecules within cells. Moreover, redox reactions have the capacity to regulate multiple enzymatic pathways in cells, which is a phenomenon referred to as redox signaling [5,6,7]. This review aims to provide a comprehensive overview of the impact of redox reactions on mitochondrial potassium (mitoK) channels. Additionally, it will describe examples of cellular redox processes wherein mitoK channels play a pivotal role. The localization of these channels within the mitochondria, in conjunction with prevalent redox reactions occurring within these organelles, offers a novel and intriguing perspective on the regulatory mechanisms governing these proteins [5,8].

### 1.1. Mitochondrial Membrane Permeability

The low permeability of the inner mitochondrial membrane (IMM) to ions, especially to protons, is the basis for maintaining mitochondrial energy homeostasis, transmembrane potential, functioning of the respiratory chain, oxidative phosphorylation (OxPhos) and overall mitochondrial efficiency [9]. The vast majority of substances, with the exception of O_2_, CO, CO_2_, NO^•^ and H_2_O_2_, among others, are unable to freely cross the inner mitochondrial membrane. In order to overcome this limitation related to the tight regulation of their transport, IMM contains a variety of membrane transport proteins that play a key role in mitochondrial functioning [10].

Potassium ions are an example of such limited diffusion, which cross the IMM barrier thanks to the presence of various potassium channels (previously known as potassium uniport) and the K^+^/H^+^ antiporter. This process is called the potassium ions cycle. In this context, the potassium ions cycle enables the coordinated flow of K^+^ between cytosol and the mitochondrial matrix, which is important for maintaining both their functional and structural homeostasis [11]. The phenomenon of potassium cycle plays a crucial role in mitochondrial functions, IMM remodeling and cell energy processes [12].

### 1.2. Mitochondrial Potassium Channels and Channels’ Pharmacology

In recent years, researchers have demonstrated that the family of mitoK channels is among the most numerous and diverse classes of mitochondrial channel proteins [13,14]. It includes calcium-activated channels such as large conductance (mitoBK_Ca_), intermediate conductance (mitoIK_Ca_), and small conductance (mitoSK_Ca_). Additionally, there are ATP-sensitive potassium (mitoK_ATP_) channels, voltage-regulated potassium (mitoKv1.3, mitoKv1.5 and mitoKv7.4) channels, mitochondrial hyperpolarization-activated cyclic nucleotide-gated (mitoHCN) channels, mitochondrial sodium-activated potassium (mitoSlo2) channels and mitochondrial two-pore domain potassium (mitoTASK) channels [14]. The activity of potassium channels undergoes changes under the influence of various external and interorganellar stimuli, such as alterations in pH, concentrations of calcium and sodium ions, temperature, ROS or the differential expression of regulatory proteins associated with potassium channels proteins [13,15,16]. It is noteworthy that potassium channels constitute not only a highly diverse group but are also widely distributed at both the cellular and tissue levels. Analogues of almost all these channels were discovered in the cell membrane and other organelles, including the endoplasmic reticulum (ER) or nucleus of various cell types [17]. 

Subtle modulation of the activity of mitoK channels affects the activity of the respiratory chain complexes. Moreover, mitoK channels also take part in the readjustment of mitochondrial matrix volume, preventing uncontrolled matrix shrinkage or swelling [18]. It has also been shown that opening of the mitoK channels has a cytoprotective effect [19,20,21]. Similar to the heart, the metabolism of brain tissue relies on the proper functioning of the respiratory chain, making it highly susceptible to hypoxia and damage during reperfusion [22]. The involvement of mitoK channels such as mitoBK_Ca_, mitoIK_Ca_, mitoK_ATP_ or mitoSK_Ca_ channels in cardio- and neuroprotective mechanisms has been demonstrated in many experimental animal models, including rats [23,24], guinea pig [25,26,27], rabbit [28,29], beef heart [30,31] and in isolated atrial trabeculae [32]. The detailed mechanism of this phenomenon will be discussed in the second section of this review. Conversely, the inhibition of mitoK channels not only reduces cell proliferation but also induces cell death [17,33,34]. The well-known mitoBK_Ca_ channels inhibitor, paxilline, has been demonstrated to eliminate the malignancy-promoting effects in murine and human models of breast cancer [35]. Thus, mitoK channels could serve a dual role: as potential therapeutic targets for ischemia/reperfusion injuries, such as heart attacks and strokes, and in the context of anticancer therapies [36,37]. Hence, ongoing research is focused on elucidating the in vivo mechanisms that regulate the activity of mitoK channels. In order to exert an exogenous influence on mitoK channel activity, numerous research groups have continually explored novel compounds characterized by high specificity for various molecular potassium channels [38]. The existing literature already reports positive protective effects on ischemia/reperfusion (I/R) processes through the activation of mitoK_ATP_ channels by the potassium channel opener, diazoxide, and the large-conductance Ca^2+^-activated potassium (mitoBK_Ca_) channels by NS1619, a benzimidazolone analogue, and its follower NS11021, which is more specific and potent channel opener [39]. Nevertheless, it is noteworthy that these compounds exhibit limited specificity toward mitoK channels [40,41,42]. In contrast, toxins isolated from the venom of various scorpion species such as iberiotoxin or charybdotoxin inhibit the activity of this channel [43,44]. There are substances that modulate most of the detected Ca^2+^-activated mitoK channels, including mitoSK_Ca_ and mitoIK_Ca_ channels which are activated by NS309 and DCEBIO and inhibited by TRAM34 [38,45]. Additionally, the activity of mitoSK_Ca_ channels is inhibited by apamin, a component of bee venom, and the activity of mitoIK_Ca_ channels is inhibited by clotrimazole. The mitoK_ATP_ channel is activated also by isoflurane, nicorandil, and (3R,4S)-4-[(4-Chlorophenyl) (1H-imidazol-2-ylmethyl) amino]-3,4-dihydro-3-hydroxy-2,2-dimethyl-2H-1-benzopyran-6-carbonitrile monohydrochloride (BMS-191095); and it is blocked by 5-hydroxydecanoic acid (5-HD) and glibenclamide [38].

### 1.3. From Redox Homeostasis to Cellular Redox Stress 

**Figure 1:**
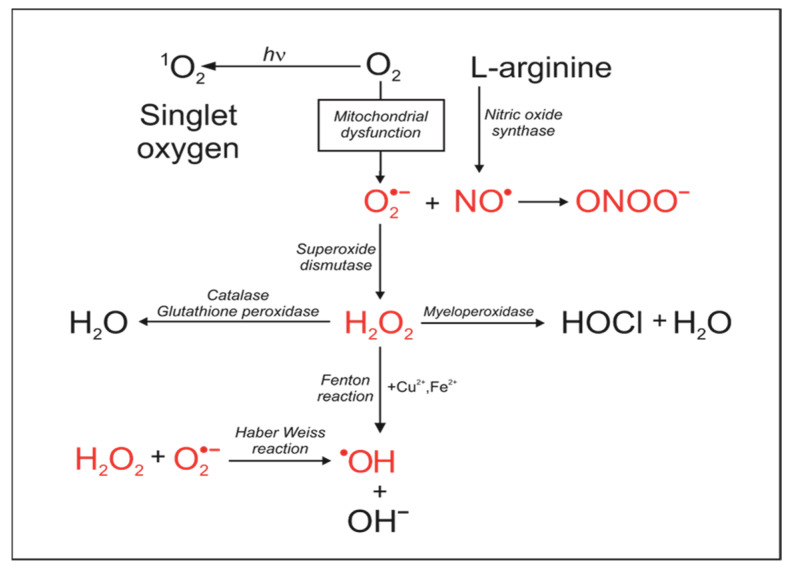
Reactions of reactive oxygen species (ROS) and reactive nitrogen species RNS. ROS, RNS and major derivatives synthesized in mitochondria: superoxide anion (O_2_^•−^); hydrogen peroxide (H_2_O_2_); nitric oxide (NO^•^), peroxynitrite (ONOO^−^); hydroxyl radical (HO^•^), hypochlorous acid (HOCl).

Reduction–oxidation (redox) reactions play a pivotal role in numerous biochemical pathways with oxygen serving as the archetypal oxidant in these intricate processes. This phenomenon is prominently exemplified in the functionality of mitochondria. It is noteworthy that redox reactions extend beyond oxygen, encompassing other essential elements such as nitrogen and sulfur [46]. The delicate balance between reducing and oxidative species gives rise to what is termed redox homeostasis, and a lack of it leads to redox stress. This imbalance is a critical aspect of cellular processes [47]. Living organisms employ a plethora of reactions to sustain their physiological functions, many of which involve the intricate interplay of oxidation and a reduction in substrates. Tretter et al. provide comprehensive insights into these processes in their publication [48]. The significance of reactive species in cellular processes regulation is well established. O-Uchi et al. present compelling evidence that the controlled production of low to moderate levels of reactive species is pivotal for the proper regulation of basic cellular processes [5]. These reactive species act as messengers, facilitating signaling mechanisms without causing undue tissue damage [48,49]. To avoid an excessive synthesis of reactive species and oxidative stress, cells have evolved cellular antioxidant enzymes and small molecules that undergo reversible oxidation. When the amount of reactive species exceeds the limit of adaptation of a cell or its compartment to the changed redox homeostasis, tissue damage occurs, which is usually in a non-specific manner. Very delicate cellular redox systems, which vary in subcellular compartments in terms of expected and safe concentrations and depending on the physiological or pathological state of cells and organelles, have emerged to cause more difficulties than previously expected in the therapeutic targeting of oxidative stress in disease states. Antioxidant enzymes catalyze the degradation of harmful oxidative species into non-reactive species, but it is also important to note that one reactive species is often converted to another with different reaction specificity (Figure 1).

**Figure 2:**
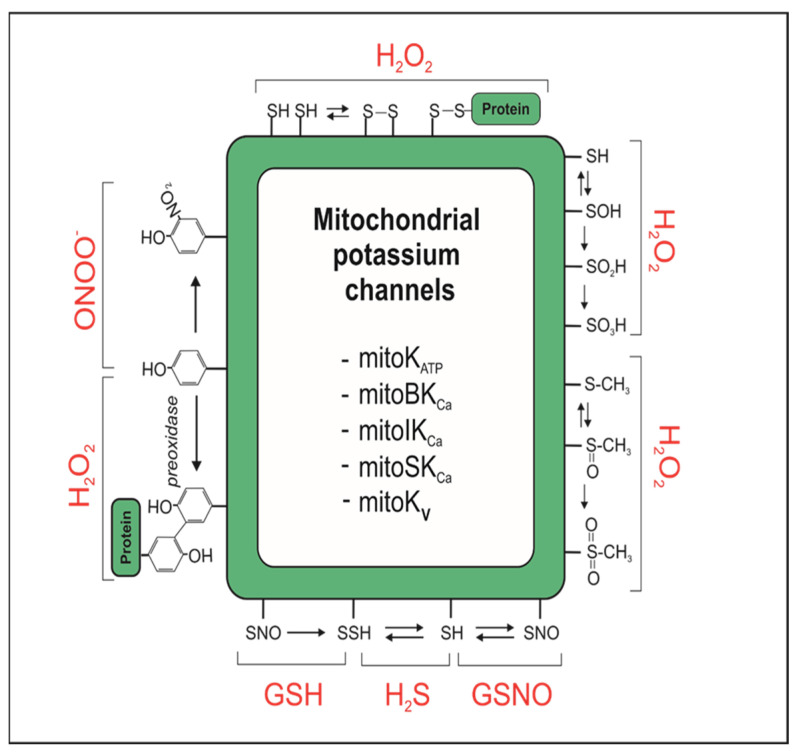
Redox modifications of mitochondrial potassium (mitoK) channels proteins. From top (clockwise): cysteine disulfide bonds formation; cysteine oxidation to sulfenic, sulfinic and sulfonic acid; methionine oxidation to sulfoxide and sulfone; cysteine S-nitrosylation (GSNO), sulfhydration (H_2_S), S-glutathionylation (GSH); dityrosine formation, tyrosine 3-nitration. Single arrow (→) means that modification is irreversible. Hydrogen sulfide (H_2_S); glutathione (GSH); S-nitrosoglutathione (GSNO); peroxynitrite (ONOO^−^). MitoK_ATP_, mitoBK_Ca_, mitoIK_Ca_, mitoSK_Ca_, mitoK_V_—different types of mitochondrial potassium (mitoK) channels. Details in text.

Mitochondrial ion channels and transporters are considered as both sensors and regulators of cellular redox signaling [5]. Potassium channels are not the exception; they are modified as other cellular proteins (Figure 2). These modifications could be signaling or pathological if the stimulus overcomes cellular hormesis. That possibility is particularly seen in an ischemia/reperfusion injury. 

## 2. Mitochondrial Potassium Channels in Ischemia/Reperfusion Injury

Ischemic heart and brain diseases are leading causes of death globally. Ischemia manifests as a restriction of blood flow to any tissue, resulting in a deficiency of oxygen necessary for cellular metabolism as well as a reduced availability of nutrients and an inadequate removal of metabolic waste products [50]. In the context of ischemic tissue damage, a more detrimental factor is the abrupt restoration of circulation and, consequently, oxygen. This leads to numerous ischemia/reperfusion (I/R) injuries. The I/R process affects various cellular components, particularly the organelles, such as mitochondria. Mitochondria play a pivotal role in initiating a cascade of events that activate intracellular processes, ultimately leading up to cellular apoptosis or necrosis [51]. Due to ischemia, OxPhos is stopped and, consequently, mitochondrial ATP synthesis is also stopped. A lack of oxygen causes an accumulation of hypoxia-inducible factor-α (HIF-1α) and causes the cell to switch to anaerobic metabolism, which is less efficient in ATP synthesis than in OxPhos [52]. Due to glycolytic metabolism, lactate is generated, leading to the acidification of the cytosol and cellular environment. Furthermore, the electron transport chain ceases to pump protons out of the matrix, resulting in a decrease in both mitochondrial membrane potential (ΔΨm) and ΔpH. The reduction in ATP concentration carries several implications. Primarily, it disrupts ion homeostasis in both mitochondria and the entire cell, as numerous systems responsible for ion homeostasis fail to function properly. Inhibiting Na^+^/K^+^-ATPase results in an elevated extracellular concentration of K^+^ and the accumulation of Na^+^ intracellularly. This elevation of Na^+^ within the cell reduces the activity of the Na^+^/Ca^2+^ exchanger. Consequently, due to the diminished electrochemical gradient, transporting Ca^2+^ outside the cell leads to Ca^2+^ overload in the cytosol and an influx of Ca^2+^ into the mitochondrial matrix [53,54]. Concerning ROS, they may be generated partially even during the ischemic period, as there is a residual amount of oxygen that remains unreduced by cytochrome c oxidase (COX). However, a significant burst of ROS occurs primarily during reperfusion, originating mainly from complex I and III. This is attributed to non-specific electron leakage and reverse electron transport (RET), respectively. The overload of mitochondria with ROS and Ca^2+^ contributes to the opening of the mitochondrial permeability transition pore (mPTP), resulting in the loss of ΔΨm [55,56,57,58]. These events are followed by apoptosis-inducing factors and cytochrome c release from mitochondria triggering cell death. Studies have demonstrated that the activation of mitoK channels exerts a cytoprotective effect, offering a potential avenue for preventing cell death. In the context of I/R, mitoK channels emerge as pivotal players and, consequently, potential therapeutic targets. The activation of mitoK channels proves instrumental in averting severe cell damage and altering cellular fate through various mechanisms. Primarily, the influx of K^+^ ions facilitated by mitoK channels induces a mild uncoupling of mitochondria. This restoration of electron flow through the respiratory chain serves to inhibit the massive formation of ROS, particularly by complex I [59,60,61]. Secondly, a moderate depolarization of IMM slows Ca^2+^ ion accumulation and prevents mPTP opening [62] averting membrane disruption and cell death signaling [18,63,64,65,66,67,68]. MitoK channels are able to react to negative phenomena occurring at several stages of ischemia/reperfusion injury (I/R injury). Since decreased ATP levels is an early symptom of an ischemic insult, the first activated mitoK channel is the mitoK_ATP_ channel, which is inhibited during normoxia. Next, the dissipation of ΔΨm could lead to the opening of the voltage-sensitive mitoKv7.4 channel [69]. Moreover, an increase in Ca^2+^ concentration leads to the activation of channels such as mitoSK_Ca_, mitoIK_Ca_ and mitoBK_Ca_. However, an increasing of Na^+^ concentration impacts the activity of the mitoSlo2 channel. Nevertheless, during the reperfusion phase, the concomitant presence of ROS may influence the activation of both mitoK_ATP_ and mitoBK_Ca_ channels [54,69,70].

## 3. Direct and Indirect Redox Regulation of Mitochondrial Potassium Channels

The mitoK channels, situated in the IMM, are exposed to reactive species generated in the mitochondrial environment. Additionally, there are reports suggesting that the activity of mitoK channels can also be subject to modification through the enzymatic action of protein kinases (see Section 3.2). As a result, the redox-induced alteration of protein kinases mediated by ROS, NO^•^ or H_2_S can significantly impact their enzymatic activity, consequently modulating the functionality of mitoK channels. 

### 3.1. Potassium Channels Regulation via Gasotransmitters: Nitric Oxide and Hydrogen Sulfide

The concept of “gasotransmitters” was introduced for the regulation of cellular function over two decades ago [71]. Since then, there has been substantial evidence demonstrating the diverse signaling effects of low concentrations of nitric oxide (NO^•^) and hydrogen sulfide (H_2_S) [72]. These effects are evident in alterations of cellular respiration, modifications in ATP synthesis, and an enhancement of cellular cytoprotection. The observed positive outcomes associated with “gasotransmitters” align with the benefits derived from the activation of numerous mitoK channels. Various research centers have concentrated their efforts on elucidating the potential connections between mitoK channels and the subtle doses of NO^•^ or H_2_S as “gasotransmitters” [73]. It has been revealed that NO^•^ and H_2_S possess the capability to directly and indirectly modulate the activity of mitoK channels (Table 1) [74]. The review above elucidated the impact of gaseous signaling molecules on the activity of mitoK channels. 

In indirect regulation, NO^•^ stimulates soluble guanylate cyclase (sGC), leading to the synthesis of cyclic guanosine monophosphate (cGMP). Subsequently, cGMP activates protein kinase G (PKG). PKG, in turn, demonstrates the capability to phosphorylate the mitoBK_Ca_ channels, thereby enhancing the probability of its opening. Compounds that elevate cGMP levels, along with ischemic preconditioning, confer cardiac protection against ischemia and reperfusion injury by modulating the activity of cardiomyocyte-specific BK_Ca_ channels [81]. 

It has additionally been postulated that a thiol/disulfide switch mechanism exists through which BK channel activity can promptly and reversibly respond to alterations in the redox state of the cell, particularly during transitions between hypoxic and normoxic conditions [82]. Second of the gasotransmitters involved in redox reactions is H_2_S, which is also endogenously produced in cells. The hydrogen sulfide influences cell bioenergetics and mitochondrial function. The effects of H_2_S depend on the dose used, with lower concentrations revealing beneficial effects, while higher concentrations manifest cytotoxicity [83,84]. In order to properly fulfill their task at physiological conditions, adequately react during stress conditions and finally adapt to a constantly changing environment, proteins including ion channels continually undergo modifications, which alter their properties and functions. Both mitoBK_Ca_ and mitoK_ATP_ may be upregulated due to S-sulfhydration by H_2_S [85,86,87]. 

The S-glutathionylation is an oxidative and reversible modification that involves the attachment of glutathione to the cysteine residue of the protein of interest [88]. This process is abundant in mitochondria. For S-glutathionylation within mitochondria, glutathione-dependent oxidoreductase 2 (GRX2) and glutathione-S-transferases (GST Alpha, Kappa, Mu, Pi and Zeta) are responsible [88,89]. However, non-enzymatic S-glutathionylation has been observed also, especially under oxidative stress, such as I/R injury. Under hypoxia, S-glutathionylation may be upregulated, leading to a hyper-glutathionylation of proteins, which activates oxidative stress signaling. On the other hand, the S-glutathionylation of mitochondrial proteins under normoxic conditions is reversible, dependent upon redox fluctuations, and an enzymatically driven modification [90]. It has been shown that enzymes of the tricarboxylic acid cycle, SLC proteins (UCP2, UCP3, ANT), all complexes of OxPhos and many other proteins within the inner mitochondrial membrane are S-glutathionylated in parallel. The S-glutathionylation of K_ATP_ has been reported. As was demonstrated by Yang and colleagues, the S-glutathionylation of a cysteine residue of the Kir6.1 channels subunit leads to K_ATP_ channels activity inhibition [91,92,93].

Given the structural similarities between plasmalemmal and mitochondrial potassium channels, the potential regulation of the mitoK_ATP_ channels [94] through S-glutathionylation appears highly plausible. Unfortunately, as of the present day, there is an absence of direct evidence to substantiate the occurrence of S-glutathionylation on mitoK_ATP_ channels.

Another group of compounds that post-translationally modified proteins in cells comprises RNS, which originate from NO^•^, including the nitroxide anion (NO^−^), nitrosonium cation (NO^+^), peroxynitrite (ONOO^−^), S-nitrosothiols, nitrogen dioxide (NO_2_), and higher nitrogen oxides. It is believed that RNS may exert a regulatory influence on physiological processes and could also contribute to the development of pathological disorders [95]. It has been demonstrated that one of the nitrox forms of RNS, namely nitroxyl (HNO), which is reduced by one electron and serves as the protonated form of NO^•^, exhibits vasoprotective and vasodilating properties. The authors propose that the vasorelaxation and vasoprotective attributed to HNO may arise from its direct modulation of sarcolemmal K_ATP_ and K_V_ channels or through the activation of sGC and cGMP [96,97,98,99,100]. It has been elucidated that not only are potassium channels located in the plasma membrane activated, but also mitoK_ATP_ channels within the inner mitochondrial membrane undergo modulation by NADPH, NO^•^, superoxide (O_2_^•−^), HNO, nitrolinoleate (LA-NO_2_), and S-nitrosothiols. Some of these compounds activate mitoK_ATP_ channels directly, such as NADPH, or indirectly through intermediaries like LA-NO_2_ and HNO, which are thought to mediate their effects via complex II. On the other hand, nitrolinoleate has also been shown to inhibit complex II, although an effective concentration of LA-NO_2_ was significantly higher than that needed for mitoK_ATP_ channels’ upregulation. It is noteworthy that during ischemic preconditioning, the concentration of LA-NO_2_ may be close to 1 μM, indicating that nitrated fatty acids, including LA-NO_2_, can influence cell survival via mitoK_ATP_ channels. The molecular mechanism remains elusive, but it is presumed that thiol groups within cysteines are the primary targets for electrophilic LA-NO_2_ [76,79,101]. It has been revealed that S-nitroso-N-acetyl-DL-penicillamine (SNAP), an NO^•^ donor, induces an increase in mitoK_ATP_ channels activity at low concentrations. However, this activation is inhibited by mitoK_ATP_ channels inhibitors such as 5-HD and glibenclamide. Conversely, high SNAP concentrations, and thus elevated NO^•^ levels, result in a decrease in mitoK_ATP_ channels activity. It is probable that heightened NO^•^ levels will inhibit cytochrome c oxidase, subsequently causing a reduction in the rate of electron flow through the respiratory chain [76,102]. 

Another oxidative protein modification is sulfhydration. As briefly mentioned earlier, sulfhydration upregulates the mitoK_ATP_ channels activity by converting the -SH group to -SSH. Additionally, this sulfhydration causes allosteric shift of the sulfonylurea receptor SUR2B subunit that enhances upregulation of potassium channel activity by its opener – levcromakalim.. Interestingly, nitration of the tyrosine residue in the Kir6.1 channel subunit was reduced as a result of sulfhydration of the SUR2B subunit. It is highly probable that C24S and C1455S within the SUR2B subunit undergo sulfhydration, as mutations in these sites prevented the cessation of nitration. This intriguing observation suggests that these protein modifications mutually influence each other within a single protein [85,103,104,105].

Mitochondria are also the main hub of reactive sulfur species (RSS), since they are places of oxidation of H_2_S. The RSS required for post-translational modification mediated by protein persulfidation are generated from mitochondria through H_2_S oxidation [106,107]. As a result of oxidation of methionine residue, the BK_Ca_ channels have been upregulated [108]. An enzyme, peptide methionine sulfoxide reductase (MsrA), which reduces methionine sulfoxides, partially abolished this effect. On the other hand, cysteine oxidation has been shown to inhibit the channel [109].

The activity of BK_Ca_ channels has been reported to be augmented by oxidants such as NAD^+^ and glutathione disulfide. Exemplify, in pulmonary arterial smooth muscle cells, 2 mM NAD^+^ and 5 mM glutathione disulfide (GSSG) have induced BK_Ca_ channels opening. On the contrary, reduced congeners—NADH and glutathione (GSH)—inhibited the channel [43,110]. Also, the sulfenylation and sulfinylation of BK_Ca_ channels have been reported. Transformation from -SOH to sulfinic (–SO_2_H) and sulfonic (–SO_3_H) acid have been shown to close the channel, considering modification to sulfonic (–SO_3_H) acid irreversibly. Nevertheless, sulfenylation can be abolished via a reducing agent able to reinstate the thiol group (-SH) in the side chain of cysteine(s), such as GSH [111,112] or dithiothreitol (DTT). The activity of BK_Ca_ channels has been shown to be downregulated also by other compounds, for example NEM, DTNB and MTSEA, whose target is the thiol group [112,113,114,115,116,117]. These data suggest that sulfhydryl groups play a key role in regulating BK_Ca_ channels. Moreover, inhibition caused by NO^•^-derived peroxynitrite in the cerebrovascular and coronary smooth muscle has been reported [118,119,120]. Finally, the redox sensitivity of BK_Ca_ channels was shown in mitoplasts obtained from mitochondria of the human endothelial cell line, EA.hy926. It has been demonstrated that a flavonoid, luteoline (LUT), has not altered mitoBK_Ca_ channels’ activity. However, in reducing conditions obtained by DTT, which is commonly known to break down disulfide bonds, LUT has activated mitoBK_Ca_ channels. This effect was hampered by the mitoBK_Ca_ channels inhibitor, paxilline, which is unexceptionable proof for mitoBK_Ca_ channels’ engagement. To our knowledge, this is the only scientific report confirming direct redox regulation of the mitoBK_Ca_ channel. Nevertheless, it could be assumed with a great dose of certainty that the mechanisms of redox regulation mentioned above also apply to the mitoBK_Ca_ channel and other congeners of BK_Ca_ channels [23,121].

### 3.2. Redox Reactions, Protein Kinases and Mitochondrial Potassium Channels Activity 

**Figure 3:**
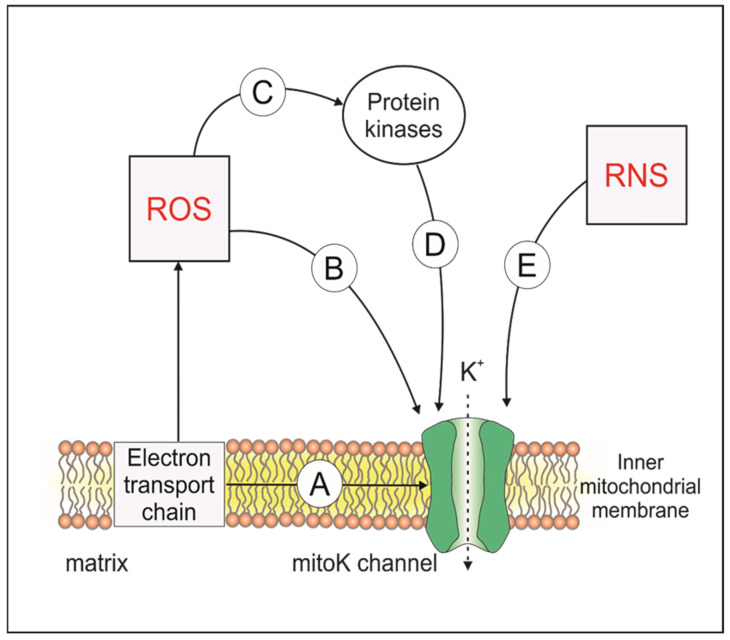
Scheme of direct and indirect regulation of mitochondrial potassium (mitoK) channels activity by electron transport chain (ETC) complexes and redox reactions. Electron transport chain (ETC) activity cause electron leak and electron interaction with oxygen to produce superoxide or hydrogen peroxide; moreover, it directly interacts with mitoK channels by protein complexes (A), the direct reaction of reactive oxygen species (ROS) with mitoK channels (B), or the ROS modification of protein kinases (PK) (C), causing an indirect regulation of mitoK channels activity by modified PK (D), while reactive nitrogen species (RNS) directly modify the mitoK channels’ activity (E).

The network of dependencies between the redox state of the cell, protein kinases activity and mitoK channels is very complex (Figure 3). Modifications of the structure and gating properties of the ion channels by their reversible phosphorylation is one of the most important mechanisms controlling their activity [70]. Some endogenous substances, like adrenomedullin, can confer cardioprotection via PKA- or PKC-mediated activation of mitoK_Ca_ or mitoK_ATP_ channels, respectively [5,122]. Since PKA and PKC enzymes are redox-sensitive kinases, the activities of both above-mentioned channels can be regulated by redox signaling [122]. For, instance some isoforms of PKC can be activated by H_2_O_2_ [123] and NO^•^ [77], which causes the higher activity of the mitoK_ATP_ channels. Interestingly, among different models that explain how diazoxide activates the K_ATP_ channel, there is also a theory that indirect activation occurs by an induction of PKC-ξ translocation [124]. PKC-ξ1 (mitochondrial PKC-ξ) is closely associated with mitoK_ATP_ channels [75]. Activation of this kinase causes opening of the mitoK_ATP_ channels. Moreover, the activator of PKC was shown to potentiate and accelerate the effect of diazoxide [16]. Potassium flux causes an increase in ROS production by complex I of the electron transport chain (ETC). These ROS activate both PKC-ξ1 (causing a positive feedback loop) and a second pool of PKC-ξ (PKCξ-2), which inhibits the mPTP. On the other hand, mitoK_ATP_ channels activation during excitotoxicity in cultured cerebellar granule neurons prevented the accumulation of ROS [125]. Interestingly, opening of the mitoK_ATP_ channels induced by PKG is not mediated by ROS [75]. Interestingly, a recent study claimed that limiting I/R injury by inhibiting the mPTP opening via PKCε activation is Independent of the mitoK_ATP_ channel [126], and some researchers doubt if mitoK_ATP_ channels exist at all [126,127]. 

## 4. Mitochondrial Potassium Channels and Respiratory Chain

The mitochondrial respiratory chain comprises a series of complex organized redox reactions, both functionally and structurally, culminating in the generation of a protonmotive force and, consequently, ATP synthesis. Certain redox centers, such as complexes I and III, can act as sources of ROS due to the release of leaky electrons, leading to the reduction in molecular oxygen. As discussed earlier, mitochondrial-generated ROS can influence the activity of mitochondrial potassium channels. There are indications proposing an alternative mechanism for the regulation of mitoK channels by the respiratory chain. Specifically, this involves the regulation of mitochondrial channels through potential interactions with respiratory chain proteins.

It is well known that mitochondrial potassium channels interact with various mitochondrial proteins [128,129,130], some of which are involved in the respiratory chain. For instance, it has been suggested that mitoK_ATP_ channels interact with succinate dehydrogenase [131,132]. In cardiac mitochondria, it was found that the β1 subunit of the mitoBK_Ca_ channels interacts with COX subunit I [133]. Furthermore, studies have demonstrated that other respiratory chain protein complexes interact with mitoBK_Ca_ channels in both cardiac [134] and brain mitochondria [135]. Additionally, mitochondrial tandem pore domain K^+^ channel TASK-3 interacts also with the respiratory chain [136]. A recent report revealed a similar interaction between the mitoKv1.3 channel and respiratory chain complex I [137]. The exact nature and functional implications of these interactions remain unclear. Is it possible that proteins comprising the respiratory chain directly interact with channel proteins, thereby regulating potassium channels activity through an unknown redox mechanism? This kind of direct functional coupling between the energy-generating system (respiratory chain) with the energy dissipation system (potassium channels) may lead to an interesting putative regulatory mechanism in mitochondria.

We found that the activity of mitoBK_Ca_ channels in glioblastoma U-87 MG cells is regulated by substrates and inhibitors of the respiratory chain [41]. This study suggested that cytochrome c oxidase (COX) is a key element of this kind of channel regulation [41]. Moreover, given that cytochrome c oxidase is the primary infrared-absorbing protein, it raises questions about the potential light regulation of mitoK channels [138]. Further research is imperative to clarify the functional consequences of these interactions. Undoubtedly, this form of regulation may prove to be distinctive and unique for mitoK channels.

## 5. Summary

Mitochondria contain the mitochondrial respiratory chain in the IMM and thus create an environment that serves as the primary source of ROS in the cell, particularly during I/R processes. Potassium channels situated in the IMM, like other proteins, undergo modification by ROS and RNS, significantly altering their activity. Studies suggest that mitoK channels not only passively encounter ROS but likely participate in numerous cellular processes. MitoK channels play a pivotal role by modulating the ΔΨm, which affects ROS synthesis, thus serving as both targets and regulators of redox reactions. Moreover, it appears that the mitoK and ROS interdependence extends; another potential site of mitoK interaction is its direct regulation of activity by respiratory chain complexes.

Interestingly, ROS play an important role in aging and lifespan [139]. Recently, it was demonstrated that BK_Ca_ channels are present in *Drosophila melanogaster* mitochondria, and channel mutants induce structural and functional defects in mitochondria, leading to an increase in ROS [140]. It also was found that the absence of BK_Ca_ channels reduced the lifespan of *Drosophila*, and the overexpression of human BK_Ca_ channels in flies extends their life. This suggested a potential role of mitochondrial potassium channels and ROS in regulating mitochondrial functional integrity and lifespan [140].

Taking into account changes in the activity of mitoK channels caused by redox reactions involving protein kinases, the regulation scheme becomes even more complex. It can be assumed that such a mechanism of regulating the activity of mitoK channels is related to subtle spatio-temporal regulation, the violation of which may lead to cell death. Furthermore, the pharmacological manipulation of mitoK channels activity through the use of inhibitors and potassium channel openers as influenced by redox reactions adds an extra layer of complexity [38].

Recent evidence strongly suggests that mitochondrial potassium channels play a significant role in inflammatory processes [141,142]. ROS synthesis in mitochondria takes place due to regulation by these proteins. It is known that mitochondria activate the inflammasome complex by releasing damage-associated molecular patterns (DAMPs) such as ROS, leading to the maturation of inflammatory molecules [141]. Additionally, inflammatory processes may be induced by an influx of potassium cations via mitoK and effects on mitochondrial structure/dynamics or effects on calcium ions overloading (followed by activation of the mitochondrial mega-channel, leading to the disruption of inner mitochondrial membrane and mitochondrial DNA release into the cytosol) [142]. 

Although the multidimensional nature of redox channel regulation presents considerable experimental challenges, it represents a crucial avenue for future research. Deciphering the complexities of redox processes in the context of mitoK channels holds promise for understanding their roles in physiological phenomena such as I/R injury, aging, inflammation, and cancer, where mitochondria play a substantial and multifaceted role.

## Figures and Tables

**Table 1:** Effect of redox species on the activity of mitoK channels.

Channel	Effect	Reactive Species	Reference
mitoK_ATP_	activation	H_2_S	[70]
		S-nitrosothiols	[75]
		nitroxyl	[76]
		nitrolinoleate	[76]
		NO	[75,76,77]
		diamide	[70,78]
		phenylarsine oxide	[70,78]
		O_2_^•−^ *	[79]
		H_2_O_2_ **	[75,76]
	inhibition	NADPH	[76]
mitoBK_Ca_	activation of the hemin-inhibited channels	H_2_S	[80]

* contrary results—Costa and Garlid (2008) [75] claim that superoxide anion was found not to open mitoK_ATP_, and superoxide-dependent opening of this channel was shown to be due to superoxide dismutation to H_2_O_2_; ** contrary results—Chiandussi et al. (2002) [78] showed that H_2_O_2_ inhibited mitoK_ATP_-dependent swelling.

## References

[1] Kakkar P., Singh B.K. (2007). Mitochondria: A Hub of Redox Activities and Cellular Distress Control. Mol. Cell. Biochem..

[2] Hatefi Y. (1985). The Mitochondrial Electron Transport and Oxidative Phosphorylation System. Annu. Rev. Biochem..

[3] Skulachev V.P., Vyssokikh M.Y., Chernyak B.V., Mulkidjanian A.Y., Skulachev M.V., Shilovsky G.A., Lyamzaev K.G., Borisov V.B., Severin F.F., Sadovnichii V.A. (2023). Six Functions of Respiration: Isn’t It Time to Take Control over ROS Production in Mitochondria, and Aging Along with It?. Int. J. Mol. Sci..

[4] Saraste M. (1999). Oxidative Phosphorylation at the Fin de Siècle. Science.

[5] O-Uchi J., Ryu S.-Y., Jhun B.S., Hurst S., Sheu S.-S. (2014). Mitochondrial Ion Channels/Transporters as Sensors and Regulators of Cellular Redox Signaling. Antioxid. Redox Signal..

[6] Di Meo S., Reed T.T., Venditti P., Victor V.M. (2016). Role of ROS and RNS Sources in Physiological and Pathological Conditions. Oxid. Med. Cell. Longev..

[7] Brookes P.S., Yoon Y., Robotham J.L., Anders M.W., Sheu S.-S. (2004). Calcium, ATP, and ROS: A Mitochondrial Love-Hate Triangle. Am. J. Physiol. Cell Physiol..

[8] Chen Q., Vazquez E.J., Moghaddas S., Hoppel C.L., Lesnefsky E.J. (2003). Production of Reactive Oxygen Species by Mitochondria: Central Role of Complex III. J. Biol. Chem..

[9] Pfanner N., Warscheid B., Wiedemann N. (2019). Mitochondrial Proteins: From Biogenesis to Functional Networks. Nat. Rev. Mol. Cell Biol..

[10] Szabo I., Zoratti M. (2014). Mitochondrial Channels: Ion Fluxes and More. Physiol. Rev..

[11] Chapa-Dubocq X.R., Rodríguez-Graciani K.M., Escobales N., Javadov S. (2023). Mitochondrial Volume Regulation and Swelling Mechanisms in Cardiomyocytes. Antioxid. Redox Signal..

[12] Garlid K.D., Paucek P. (2003). Mitochondrial Potassium Transport: The K^+^ Cycle. Biochim. Et Biophys. Acta (BBA)-Bioenerg..

[13] O’Rourke B. (2007). Mitochondrial Ion Channels. Annu. Rev. Physiol..

[14] Szabo I., Szewczyk A. (2023). Mitochondrial Ion Channels. Annu. Rev. Biophys..

[15] Debska G., Kicińska A., Skalska J., Szewczyk A. (2001). Intracellular Potassium and Chloride Channels: An Update. Acta Biochim. Pol..

[16] Kicińska A., Debska G., Kunz W., Szewczyk A. (2000). Mitochondrial Potassium and Chloride Channels. Acta Biochim. Pol..

[17] Checchetto V., Leanza L., De Stefani D., Rizzuto R., Gulbins E., Szabo I. (2021). Mitochondrial K^+^ Channels and Their Implications for Disease Mechanisms. Pharmacol. Ther..

[18] Laskowski M., Augustynek B., Kulawiak B., Koprowski P., Bednarczyk P., Jarmuszkiewicz W., Szewczyk A. (2016). What Do We Not Know about Mitochondrial Potassium Channels?. Biochim. Et Biophys. Acta (BBA)-Bioenerg..

[19] O’Rourke B. (2004). Evidence for Mitochondrial K^+^ Channels and Their Role in Cardioprotection. Circ. Res..

[20] Kulawiak B., Kudin A.P., Szewczyk A., Kunz W.S. (2008). BK Channel Openers Inhibit ROS Production of Isolated Rat Brain Mitochondria. Exp. Neurol..

[21] Szewczyk A., Marbán E. (1999). Mitochondria: A New Target for K Channel Openers?. Trends Pharmacol. Sci..

[22] Pérez-Pinzón M.A. (2004). Neuroprotective Effects of Ischemic Preconditioning in Brain Mitochondria Following Cerebral Ischemia. J. Bioenerg. Biomembr..

[23] Kampa R.P., Flori L., Sęk A., Spezzini J., Brogi S., Szewczyk A., Calderone V., Bednarczyk P., Testai L. (2022). Luteolin-Induced Activation of Mitochondrial BK_Ca_ Channels: Undisclosed Mechanism of Cytoprotection. Antioxid. Redox Signal..

[24] Liang H.-W., Xia Q., Bruce I.C. (2005). Reactive Oxygen Species Mediate the Neuroprotection Conferred by a Mitochondrial ATP-Sensitive Potassium Channel Opener during Ischemia in the Rat Hippocampal Slice. Brain Res..

[25] Zhu Z., Li Y.L., Li D.P., He R.R. (2000). Effect of Anoxic Preconditioning on ATP-Sensitive Potassium Channels in Guinea-Pig Ventricular Myocytes. Pflügers Arch..

[26] Xu W., Liu Y., Wang S., McDonald T., Van Eyk J.E., Sidor A., O’Rourke B. (2002). Cytoprotective Role of Ca^2+^- Activated K^+^ Channels in the Cardiac Inner Mitochondrial Membrane. Science.

[27] Yang M., Camara A.K.S., Aldakkak M., Kwok W.M., Stowe D.F. (2017). Identity and Function of a Cardiac Mitochondrial Small Conductance Ca^2+^-Activated K^+^ Channel Splice Variant. Biochim. Biophys. Acta Bioenerg..

[28] Nakai Y., Horimoto H., Mieno S., Sasaki S. (2001). Mitochondrial ATP-Sensitive Potassium Channel Plays a Dominant Role in Ischemic Preconditioning of Rabbit Heart. Eur. Surg. Res..

[29] Bai Y., Muqier, Murakami H., Iwasa M., Sumi S., Yamada Y., Ushikoshi H., Aoyama T., Nishigaki K., Takemura G. (2011). Cilostazol Protects the Heart against Ischaemia Reperfusion Injury in a Rabbit Model of Myocardial Infarction: Focus on Adenosine, Nitric Oxide and Mitochondrial ATP-Sensitive Potassium Channels. Clin. Exp. Pharmacol. Physiol..

[30] Paucek P., Mironova G., Mahdi F., Beavis A.D., Woldegiorgis G., Garlid K.D. (1992). Reconstitution and Partial Purification of the Glibenclamide-Sensitive, ATP-Dependent K^+^ Channel from Rat Liver and Beef Heart Mitochondria. J. Biol. Chem..

[31] Bednarczyk P., Kicińska A., Kominkova V., Ondrias K., Dolowy K., Szewczyk A. (2004). Quinine Inhibits Mitochondrial ATP-Regulated Potassium Channel from Bovine Heart. J. Membr. Biol..

[32] Pomerantz B.J., Robinson T.N., Morrell T.D., Heimbach J.K., Banerjee A., Harken A.H. (2000). Selective Mitochondrial Adenosine Triphosphate–sensitive Potassium Channel Activation Is Sufficient to Precondition Human Myocardium. J. Thorac. Cardiovasc. Surg..

[33] Bachmann M., Pontarin G., Szabo I. (2019). The Contribution of Mitochondrial Ion Channels to Cancer Development and Progression. Cell. Physiol. Biochem..

[34] Checchetto V., Azzolini M., Peruzzo R., Capitanio P., Leanza L. (2018). Mitochondrial Potassium Channels in Cell Death. Biochem. Biophys. Res. Commun..

[35] Bischof H., Maier S., Koprowski P., Kulawiak B., Burgstaller S., Jasińska J., Serafimov K., Gross D., Schroth W., Matt L. (2023). MitoBKCa Is Functionally Expressed in Murine and Human Breast Cancer Cells and Promotes Metabolic Reprogramming. eLife.

[36] Leanza L., Venturini E., Kadow S., Carpinteiro A., Gulbins E., Becker K.A. (2015). Targeting a Mitochondrial Potassium Channel to Fight Cancer. Cell Calcium.

[37] Szabo I., Zoratti M., Biasutto L. (2021). Targeting Mitochondrial Ion Channels for Cancer Therapy. Redox Biol..

[38] Augustynek B., Kunz W.S., Szewczyk A., Singh H., Sheu S.-S. (2017). Guide to the Pharmacology of Mitochondrial Potassium Channels. Pharmacology of Mitochondria.

[39] Bentzen B.H., Nardi A., Calloe K., Madsen L.S., Olesen S.-P., Grunnet M. (2007). The Small Molecule NS11021 Is a Potent and Specific Activator of Ca^2+^-Activated Big-Conductance K^+^ Channels. Mol. Pharmacol..

[40] Siemen D., Loupatatzis C., Borecky J., Gulbins E., Lang F. (1999). Ca^2+^-Activated K Channel of the BK-Type in the Inner Mitochondrial Membrane of a Human Glioma Cell Line. Biochem. Biophys. Res. Commun..

[41] Bednarczyk P., Wieckowski M.R., Broszkiewicz M., Skowronek K., Siemen D., Szewczyk A. (2013). Putative Structural and Functional Coupling of the Mitochondrial BK Channel to the Respiratory Chain. PLoS ONE.

[42] Wrzosek A., Gałecka S., Żochowska M., Olszewska A., Kulawiak B. (2022). Alternative Targets for Modulators of Mitochondrial Potassium Channels. Molecules.

[43] Park C.S., Miller C. (1992). Mapping Function to Structure in a Channel-Blocking Peptide: Electrostatic Mutants of Charybdotoxin. Biochemistry.

[44] Candia S., Garcia M.L., Latorre R. (1992). Mode of Action of Iberiotoxin, a Potent Blocker of the Large Conductance Ca^2+^-Activated K^+^ Channel. Biophys. J..

[45] Sun W.-T., Xue H.-M., Hou H.-T., Chen H.-X., Wang J., He G.-W., Yang Q. (2021). Homocysteine Alters Vasoreactivity of Human Internal Mammary Artery by Affecting the K Channel Family. Ann. Transl. Med..

[46] Kabil O., Motl N., Banerjee R. (2014). H_2_S and Its Role in Redox Signaling. Biochim. Et Biophys. Acta (BBA)-Proteins Proteom..

[47] Ruhland B.R., Reniere M.L. (2019). Sense and Sensor Ability: Redox-Responsive Regulators in Listeria Monocytogenes. Curr. Opin. Microbiol..

[48] Tretter V., Hochreiter B., Zach M.L., Krenn K., Klein K.U. (2022). Understanding Cellular Redox Homeostasis: A Challenge for Precision Medicine. Int. J. Mol. Sci..

[49] Forman H.J., Zhang H. (2021). Targeting Oxidative Stress in Disease: Promise and Limitations of Antioxidant Therapy. Nat. Rev. Drug Discov..

[50] Kalogeris T., Baines C.P., Krenz M., Korthuis R.J. (2012). Cell Biology of Ischemia/reperfusion Injury. Int. Rev. Cell Mol. Biol..

[51] Honda H.M., Korge P., Weiss J.N. (2005). Mitochondria and Ischemia/reperfusion Injury. Ann. N. Y. Acad. Sci..

[52] Rey S., Semenza G.L. (2010). Hypoxia-Inducible Factor-1-Dependent Mechanisms of Vascularization and Vascular Remodelling. Cardiovasc. Res..

[53] Kadenbach B., Ramzan R., Moosdorf R., Vogt S. (2011). The Role of Mitochondrial Membrane Potential in Ischemic Heart Failure. Mitochondrion.

[54] Kulawiak B., Szewczyk A. (2022). Current Challenges of Mitochondrial Potassium Channel Research. Front. Physiol..

[55] Di Lisa F., Bernardi P. (2006). Mitochondria and Ischemia-Reperfusion Injury of the Heart: Fixing a Hole. Cardiovasc. Res..

[56] Halestrap A.P., Pasdois P. (2009). The Role of the Mitochondrial Permeability Transition Pore in Heart Disease. Biochim. Biophys. Acta.

[57] Halestrap A.P. (2009). Mitochondrial Calcium in Health and Disease. Biochim. Biophys. Acta.

[58] Hausenloy D.J., Yellon D.M. (2007). Reperfusion Injury Salvage Kinase Signalling: Taking a RISK for Cardioprotection. Heart Fail. Rev..

[59] Krabbendam I.E., Honrath B., Culmsee C., Dolga A.M. (2018). Mitochondrial Ca^2+^-Activated K^+^ Channels and Their Role in Cell Life and Death Pathways. Cell Calcium.

[60] Malinska D., Mirandola S.R., Kunz W.S. (2010). Mitochondrial Potassium Channels and Reactive Oxygen Species. FEBS Lett..

[61] Szewczyk A., Kajma A., Malinska D., Wrzosek A., Bednarczyk P., Zabłocka B., Dołowy K. (2010). Pharmacology of Mitochondrial Potassium Channels: Dark Side of the Field. FEBS Lett..

[62] Korge P., Honda H.M., Weiss J.N. (2002). Protection of Cardiac Mitochondria by Diazoxide and Protein Kinase C: Implications for Ischemic Preconditioning. Proc. Natl. Acad. Sci. USA.

[63] Krabbendam I. (2020). The Role of Small Conductance Calcium-Activated Potassium Channels in Mitochondrial Dysfunction: Targeting Metabolic Reprogramming and Calcium Homeostasis. Ph.D. Thesis.

[64] Urbani A., Prosdocimi E., Carrer A., Checchetto V., Szabò I. (2021). Mitochondrial Ion Channels of the Inner Membrane and Their Regulation in Cell Death Signaling. Front. Cell Dev. Biol..

[65] Sánchez E.C. (2019). Pathophysiology of Ischemia-Reperfusion Injury and Its Management with Hyperbaric Oxygen (HBO): A Review. J. Emerg. Crit. Care Med..

[66] Behera R., Sharma V., Grewal A.K., Kumar A., Arora B., Najda A., Albadrani G.M., Altyar A.E., Abdel-Daim M.M., Singh T.G. (2023). Mechanistic Correlation between Mitochondrial Permeability Transition Pores and Mitochondrial ATP Dependent Potassium Channels in Ischemia Reperfusion. Biomed. Pharmacother..

[67] Jarmuszkiewicz W., Szewczyk A. (2019). Energy-Dissipating Hub in Muscle Mitochondria: Potassium Channels and Uncoupling Proteins. Arch. Biochem. Biophys..

[68] Borutaite V., Toleikis A., Brown G.C. (2013). In the Eye of the Storm: Mitochondrial Damage during Heart and Brain Ischaemia. FEBS J..

[69] Kulawiak B., Bednarczyk P., Szewczyk A. (2021). Multidimensional Regulation of Cardiac Mitochondrial Potassium Channels. Cells.

[70] Rotko D., Kunz W.S., Szewczyk A., Kulawiak B. (2020). Signaling Pathways Targeting Mitochondrial Potassium Channels. Int. J. Biochem. Cell Biol..

[71] Wang R. (2003). The Gasotransmitter Role of Hydrogen Sulfide. Antioxid. Redox Signal..

[72] Wang R. (2004). Signal Transduction and the Gasotransmitters: NO, CO, and H_2_S in Biology and Medicine.

[73] Gessner G., Sahoo N., Swain S.M., Hirth G., Schönherr R., Mede R., Westerhausen M., Brewitz H.H., Heimer P., Imhof D. (2017). CO-Independent Modification of K^+^ Channels by tricarbonyldichlororuthenium(II) Dimer (CORM-2). Eur. J. Pharmacol..

[74] Walewska A., Szewczyk A., Koprowski P. (2018). Gas Signaling Molecules and Mitochondrial Potassium Channels. Int. J. Mol. Sci..

[75] Costa A.D.T., Garlid K.D. (2008). Intramitochondrial Signaling: Interactions among mitoK_ATP_, PKCε, ROS, and MPT. Am. J. Physiol. -Heart Circ. Physiol..

[76] Queliconi B.B., Wojtovich A.P., Nadtochiy S.M., Kowaltowski A.J., Brookes P.S. (2011). Redox Regulation of the Mitochondrial K_ATP_ Channel in Cardioprotection. Biochim. Biophys. Acta.

[77] Harada N., Miura T., Dairaku Y., Kametani R., Shibuya M., Wang R., Kawamura S., Matsuzaki M. (2004). NO Donor-Activated PKC-δ Plays a Pivotal Role in Ischemic Myocardial Protection through Accelerated Opening of Mitochondrial K-ATP Channels. J. Cardiovasc. Pharmacol..

[78] Chiandussi E., Petrussa E., Macrì F., Vianello A. (2002). Modulation of a Plant Mitochondrial K^+^ATP Channel and Its Involvement in Cytochrome c Release. J. Bioenerg. Biomembr..

[79] Zhang D.X., Chen Y.-F., Campbell W.B., Zou A.-P., Gross G.J., Li P.-L. (2001). Characteristics and Superoxide-Induced Activation of Reconstituted Myocardial Mitochondrial ATP-Sensitive Potassium Channels. Circ. Res..

[80] Walewska A., Szewczyk A., Krajewska M., Koprowski P. (2022). Targeting Mitochondrial Large-Conductance Calcium-Activated Potassium Channel by Hydrogen Sulfide via Heme-Binding Site. J. Pharmacol. Exp. Ther..

[81] Frankenreiter S., Bednarczyk P., Kniess A., Bork N.I., Straubinger J., Koprowski P., Wrzosek A., Mohr E., Logan A., Murphy M.P. (2017). cGMP-Elevating Compounds and Ischemic Conditioning Provide Cardioprotection Against Ischemia and Reperfusion Injury via Cardiomyocyte-Specific BK Channels. Circulation.

[82] Yi L., Morgan J.T., Ragsdale S.W. (2010). Identification of a Thiol/disulfide Redox Switch in the Human BK Channel That Controls Its Affinity for Heme and CO. J. Biol. Chem..

[83] Paul B.D., Snyder S.H., Kashfi K. (2021). Effects of Hydrogen Sulfide on Mitochondrial Function and Cellular Bioenergetics. Redox Biol..

[84] Łoboda A., Dulak J. (2024). Cardioprotective Effects of Hydrogen Sulfide and Its Potential Therapeutic Implications in the Amelioration of Duchenne Muscular Dystrophy Cardiomyopathy. Cells.

[85] Iciek M., Kowalczyk-Pachel D., Bilska-Wilkosz A., Kwiecień I., Górny M., Włodek L. (2016). S-Sulfhydration as a Cellular Redox Regulation. Biosci. Rep..

[86] Liang W., Chen J., Mo L., Ke X., Zhang W., Zheng D., Pan W., Wu S., Feng J., Song M. (2016). ATP-Sensitive K^+^ Channels Contribute to the Protective Effects of Exogenous Hydrogen Sulfide against High Glucose-Induced Injury in H9c2 Cardiac Cells. Int. J. Mol. Med..

[87] Flori L., Montanaro R., Pagnotta E., Ugolini L., Righetti L., Martelli A., Di Cesare Mannelli L., Ghelardini C., Brancaleone V., Testai L. (2023). Erucin Exerts Cardioprotective Effects on Ischemia/Reperfusion Injury through the Modulation of mitoKATP Channels. Biomedicines.

[88] Vrettou S., Wirth B. (2022). S-Glutathionylation and S-Nitrosylation in Mitochondria: Focus on Homeostasis and Neurodegenerative Diseases. Int. J. Mol. Sci..

[89] Goto S., Kawakatsu M., Izumi S., Urata Y., Kageyama K., Ihara Y., Koji T., Kondo T. (2009). Glutathione S-Transferase Pi Localizes in Mitochondria and Protects against Oxidative Stress. Free Radic. Biol. Med..

[90] Shemarova I., Nesterov V., Emelyanova L., Korotkov S. (2021). Mitochondrial Mechanisms by Which Gasotransmitters (H_2_S, NO and CO) Protect Cardiovascular System against Hypoxia. Front. Biosci..

[91] Yang Y., Shi W., Cui N., Wu Z., Jiang C. (2010). Oxidative Stress Inhibits Vascular KATP Channels by S-Glutathionylation. J. Biol. Chem..

[92] Yang Y., Shi W., Chen X., Cui N., Konduru A.S., Shi Y., Trower T.C., Zhang S., Jiang C. (2011). Molecular Basis and Structural Insight of Vascular K_ATP_ Channel Gating by S-Glutathionylation. J. Biol. Chem..

[93] Weise-Cross L., Resta T.C., Jernigan N.L. (2019). Redox Regulation of Ion Channels and Receptors in Pulmonary Hypertension. Antioxid. Redox Signal..

[94] Peng K., Hu J., Xiao J., Dan G., Yang L., Ye F., Zou Z., Cao J., Sai Y. (2018). Mitochondrial ATP-Sensitive Potassium Channel Regulates Mitochondrial Dynamics to Participate in Neurodegeneration of Parkinson’s Disease. Biochim. Et Biophys. Acta (BBA)-Mol. Basis Dis..

[95] Zhou Y., Yang X., Zhang J., Xu S., Li J., Wang W., Yan M. (2023). Small Molecule Fluorescent Probes for the Detection of Reactive Nitrogen Species in Biological Systems. Coord. Chem. Rev..

[96] Favaloro J.L., Kemp-Harper B.K. (2007). The Nitroxyl Anion (HNO) Is a Potent Dilator of Rat Coronary Vasculature. Cardiovasc. Res..

[97] Favaloro J.L., Kemp-Harper B.K. (2009). Redox Variants of NO (NO^•^ and HNO) Elicit Vasorelaxation of Resistance Arteries via Distinct Mechanisms. Am. J. Physiol. Heart Circ. Physiol..

[98] Bullen M.L., Miller A.A., Andrews K.L., Irvine J.C., Ritchie R.H., Sobey C.G., Kemp-Harper B.K. (2011). Nitroxyl (HNO) as a Vasoprotective Signaling Molecule. Antioxid. Redox Signal..

[99] Zarpelon A.C., Souza G.R., Cunha T.M., Schivo I.R., Marchesi M., Casagrande R., Pinge-Filho P., Cunha F.Q., Ferreira S.H., Miranda K.M. (2013). The Nitroxyl Donor, Angeli’s Salt, Inhibits Inflammatory Hyperalgesia in Rats. Neuropharmacology.

[100] Irvine J.C., Favaloro J.L., Kemp-Harper B.K. (2003). NO^−^ Activates Soluble Guanylate Cyclase and K_v_ Channels to Vasodilate Resistance Arteries. Hypertension.

[101] Nadtochiy S.M., Baker P.R.S., Freeman B.A., Brookes P.S. (2008). Mitochondrial Nitroalkene Formation and Mild Uncoupling in Ischaemic Preconditioning: Implications for Cardioprotection. Cardiovasc. Res..

[102] Brunori M., Giuffrè A., Forte E., Mastronicola D., Barone M.C., Sarti P. (2004). Control of Cytochrome c Oxidase Activity by Nitric Oxide. Biochim. Et Biophys. Acta (BBA)-Bioenerg..

[103] Sanchez L.D., Sanchez-Aranguren L., Marwah M., Wang K., Spickett C.M., Griffiths H.R., Dias I.H.K. (2022). Exploring Mitochondrial Hydrogen Sulfide Signalling for Therapeutic Interventions in Vascular Diseases. Adv. Redox Res..

[104] Kang M., Hashimoto A., Gade A., Akbarali H.I. (2015). Interaction between Hydrogen Sulfide-Induced Sulfhydration and Tyrosine Nitration in the K_ATP_ Channel Complex. Am. J. Physiol. Gastrointest. Liver Physiol..

[105] Jiang B., Tang G., Cao K., Wu L., Wang R. (2010). Molecular Mechanism for H_2_S-Induced Activation of K_ATP_ Channels. Antioxid. Redox Signal..

[106] Zhou L., Wang Q. (2023). Advances of H_2_S in Regulating Neurodegenerative Diseases by Preserving Mitochondria Function. Antioxid. Redox Signal..

[107] Kimura H. (2015). Signaling Molecules: Hydrogen Sulfide and Polysulfide. Antioxid. Redox Signal..

[108] Sobey C.G., Heistad D.D., Faraci F.M. (1997). Mechanisms of Bradykinin-Induced Cerebral Vasodilatation in Rats. Evidence That Reactive Oxygen Species Activate K^+^ Channels. Stroke.

[109] Tang X.D., Daggett H., Hanner M., Garcia M.L., McManus O.B., Brot N., Weissbach H., Heinemann S.H., Hoshi T. (2001). Oxidative Regulation of Large Conductance Calcium-Activated Potassium Channels. J. Gen. Physiol..

[110] Lee S., Park M., So I., Earm Y.E. (1994). NADH and NAD Modulates Ca^2+^-Activated K^+^ Channels in Small Pulmonary Arterial Smooth Muscle Cells of the Rabbit. Pflügers Arch..

[111] Brakemeier S., Eichler I., Knorr A., Fassheber T., Köhler R., Hoyer J. (2003). Modulation of Ca^2+^-Activated K^+^ Channel in Renal Artery Endothelium in Situ by Nitric Oxide and Reactive Oxygen Species. Kidney Int..

[112] DiChiara T.J., Reinhart P.H. (1997). Redox Modulation of Hslo Ca^2+^-Activated K^+^ Channels. J. Neurosci..

[113] Wang Z.-W., Nara M., Wang Y.-X., Kotlikoff M.I. (1997). Redox Regulation of Large Conductance Ca^2+^-Activated K^+^ Channels in Smooth Muscle Cells. J. Gen. Physiol..

[114] Tang X.D., Garcia M.L., Heinemann S.H., Hoshi T. (2004). Reactive Oxygen Species Impair Slo1 BK Channel Function by Altering Cysteine-Mediated Calcium Sensing. Nat. Struct. Mol. Biol..

[115] Hou S., Heinemann S.H., Hoshi T. (2009). Modulation of BK_Ca_ Channel Gating by Endogenous Signaling Molecules. Physiology.

[116] Hou S., Horrigan F.T., Xu R., Heinemann S.H., Hoshi T. (2009). Comparative Effects of H^+^ and Ca^2+^ on Large-Conductance Ca^2+^- and Voltage-Gated Slo1 K^+^ Channels. Channels.

[117] Murphy M.P. (2012). Mitochondrial Thiols in Antioxidant Protection and Redox Signaling: Distinct Roles for Glutathionylation and Other Thiol Modifications. Antioxid. Redox Signal..

[118] Brzezinska A.K., Gebremedhin D., Chilian W.M., Kalyanaraman B., Elliott S.J. (2000). Peroxynitrite Reversibly Inhibits Ca^2+^-Activated K^+^ Channels in Rat Cerebral Artery Smooth Muscle Cells. Am. J. Physiol. Heart Circ. Physiol..

[119] Liu Y., Gutterman D.D. (2002). Oxidative Stress and Potassium Channel Function. Clin. Exp. Pharmacol. Physiol..

[120] Liu Y., Terata K., Chai Q., Li H., Kleinman L.H., Gutterman D.D. (2002). Peroxynitrite Inhibits Ca^2+^-Activated K^+^ Channel Activity in Smooth Muscle of Human Coronary Arterioles. Circ. Res..

[121] Alliegro M.C. (2000). Effects of Dithiothreitol on Protein Activity Unrelated to Thiol–Disulfide Exchange: For Consideration in the Analysis of Protein Function with Cleland’s Reagent. Anal. Biochem..

[122] Nishida H., Sato T., Ogura T., Nakaya H. (2009). New Aspects for the Treatment of Cardiac Diseases Based on the Diversity of Functional Controls on Cardiac Muscles: Mitochondrial Ion Channels and Cardioprotection. J. Pharmacol. Sci..

[123] Genestra M. (2007). Oxyl Radicals, Redox-Sensitive Signalling Cascades and Antioxidants. Cell. Signal..

[124] Wrzosek A., Augustynek B., Żochowska M., Szewczyk A. (2020). Mitochondrial Potassium Channels as Druggable Targets. Biomolecules.

[125] Fornazari M., de Paula J.G., Castilho R.F., Kowaltowski A.J. (2008). Redox Properties of the Adenoside Triphosphate-Sensitive K^+^ Channel in Brain Mitochondria. J. Neurosci. Res..

[126] Mancardi D., Pagliaro P., Ridnour L.A., Tocchetti C.G., Miranda K., Juhaszova M., Sollott S.J., Wink D.A., Paolocci N. (2022). HNO Protects the Myocardium against Reperfusion Injury, Inhibiting the mPTP Opening via PKCε Activation. Antioxidants.

[127] Garlid K.D., Halestrap A.P. (2012). The Mitochondrial K_ATP_ Channel--Fact or Fiction?. J. Mol. Cell. Cardiol..

[128] Kathiresan T., Harvey M., Orchard S., Sakai Y., Sokolowski B. (2009). A Protein Interaction Network for the Large Conductance Ca^2+^-Activated K^+^ Channel in the Mouse Cochlea. Mol. Cell. Proteom..

[129] Peng Z., Sakai Y., Kurgan L., Sokolowski B., Uversky V. (2014). Intrinsic Disorder in the BK Channel and Its Interactome. PLoS ONE.

[130] Sokolowski B., Orchard S., Harvey M., Sridhar S., Sakai Y. (2011). Conserved BK Channel-Protein Interactions Reveal Signals Relevant to Cell Death and Survival. PLoS ONE.

[131] Ardehali H., Chen Z., Ko Y., Mejia-Alvarez R., Marban E. (2004). Multiprotein Complex Containing Succinate Dehydrogenase Confers Mitochondrial ATP-Sensitive K^+^ Channel Activity. Proc. Natl. Acad. Sci. USA.

[132] Wojtovich A.P., Williams D.M., Karcz M.K., Lopes C.M., Gray D.A., Nehrke K.W., Brookes P.S. (2010). A Novel Mitochondrial K_ATP_ Channel Assay. Circ. Res..

[133] Ohya S., Kuwata Y., Sakamoto K., Muraki K., Imaizumi Y. (2005). Cardioprotective Effects of Estradiol Include the Activation of Large-Conductance Ca^2+^-Activated K^+^ Channels in Cardiac Mitochondria. Am. J. Physiol.-Heart Circ. Physiol..

[134] Zhang J., Li M., Zhang Z., Zhu R., Olcese R., Stefani E., Toro L. (2017). The Mitochondrial BK_Ca_ Channel Cardiac Interactome Reveals BK_Ca_ Association with the Mitochondrial Import Receptor Subunit Tom22, and the Adenine Nucleotide Translocator. Mitochondrion.

[135] Singh H., Li M., Hall L., Chen S., Sukur S., Lu R., Caputo A., Meredith A.L., Stefani E., Toro L. (2016). MaxiK Channel Interactome Reveals Its Interaction with GABA Transporter 3 and Heat Shock Protein 60 in the Mammalian Brain. Neuroscience.

[136] Yao J., McHedlishvili D., McIntire W.E., Guagliardo N.A., Erisir A., Coburn C.A., Santarelli V.P., Bayliss D.A., Barrett P.Q. (2017). Functional TASK-3-Like Channels in Mitochondria of Aldosterone-Producing Zona Glomerulosa Cells. Hypertension.

[137] Peruzzo R., Mattarei A., Azzolini M., Becker-Flegler K.A., Romio M., Rigoni G., Carrer A., Biasutto L., Parrasia S., Kadow S. (2020). Insight into the Mechanism of Cytotoxicity of Membrane-Permeant Psoralenic Kv1.3 Channel Inhibitors by Chemical Dissection of a Novel Member of the Family. Redox Biol..

[138] Szewczyk A., Bednarczyk P. (2018). Modulation of the Mitochondrial Potassium Channel Activity by Infrared Light. Biophys. J..

[139] Karagianni C., Bazopoulou D. (2024). Redox Regulation in Lifespan Determination. J. Biol. Chem..

[140] Gururaja Rao S., Bednarczyk P., Towheed A., Shah K., Karekar P., Ponnalagu D., Jensen H.N., Addya S., Reyes B.A.S., Van Bockstaele E.J. (2019). BK_Ca_ (Slo) Channel Regulates Mitochondrial Function and Lifespan in Drosophila Melanogaster. Cells.

[141] Strickland M., Yacoubi-Loueslati B., Bouhaouala-Zahar B., Pender S.L.F., Larbi A. (2019). Relationships Between Ion Channels, Mitochondrial Functions and Inflammation in Human Aging. Front. Physiol..

[142] Pain P., Spinelli F., Gherardi G. (2023). Mitochondrial Cation Signalling in the Control of Inflammatory Processes. Int. J. Mol. Sci..

